# Effects of controlled hypotension with restrictive transfusion on intraoperative blood loss and systemic oxygen metabolism in elderly patients who underwent lumbar fusion

**DOI:** 10.1186/s13063-020-05015-5

**Published:** 2021-01-28

**Authors:** Xiaodong Qiu, Zhiying Tan, Wenhao Tang, Hui Ye, Xinjian Lu

**Affiliations:** 1 Department of Anesthesiology, Zhongda Hospital, Medical School, Southeast University, Nanjing, China; 2 Department of General Surgery, Zhongda Hospital of Southeast University, No. 87 Dingjiaqiao, Nanjing, 210009 China

**Keywords:** Controlled hypotension, Restrictive transfusion, Systemic oxygen metabolism, Renal function

## Abstract

**Background:**

The effects of restrictive fluid therapy combined with controlled hypotension in the elderly on systemic oxygen metabolism and renal function are clinical concerns. The aim of this study was to evaluate blood loss, oxygen metabolism, and renal function in different levels of controlled hypotension induced by intravenous nitroglycerin, in combination with limited infusion, in elderly patients undergoing posterior lumbar fusion.

**Methods:**

A total of 40 patients, aged 60–75 with ASA grade II or III, who were planned for posterior lumbar fusion were randomly allocated into two groups: experimental group [target mean arterial pressure 65 mmHg (MAP 65) or control group (MAP 75)]. Indicators for blood loss, hemodynamic, systemic oxygen metabolism, and renal function evaluation index were recorded before operation (T0), 1 h after induced hypotension (T1), 2 h after hypotension (T2), and in recovery (T3). We compared changes in these parameters between groups to evaluate the combined effects of controlled hypotension with restrictive infusion.

**Results:**

CI, DO_2_I, and VO_2_I were lower in both groups at T1–T3 compared with T0 (*p* < 0.05). DO_2_I and VO_2_I in the MAP 65 group were lower than the MAP 75 group after operation. In both groups, SCysC increased at T1, T2, and T3 (*p* < 0.05) compared with T0.

**Conclusions:**

Restrictive transfusion and control MAP at 65 mmHg can slightly change in renal function and reduce the risk of insufficient oxygen supply and importantly have no significant effect on blood loss and postoperative complications.

**Trial registration:**

ChiCTR-INR-16008153. Registered on 25 March 2016.

## Background

Spondylosis, also known as degenerative disk diseases (DDD), refers to age-related degeneration of the spine motions segments, including vertebrae, facet joints, and intervertebral disks [1]. Clinical manifestation of lumbar spondylosis has been studied extensively, and the average incidence rate was reported at approximately 4.2/1000 person-years [2]. The condition affects people of wide-range ages with a peak incidence at approximately 40 [3], usually aggravated with increased age [4].

Posterior lumbar fusion is the main surgical procedure for treatment of spinal degenerative diseases [5]. Due to complex anatomy, the surgery is often complicated with trauma, long operation time, and significant bleeding [6]. Bleeding during operation not only affects the clarity of operation field which therefore may prolong operation, but also increases the risk of requirement of allogeneic blood transfusion, which could hinder postoperative rehabilitation [7]. Controlled hypotensive anesthesia is one of the most effective methods to reduce bleeding during surgery [8]. At present, the advantages of controlled hypotension in reducing intraoperative blood loss in spinal surgery have been widely recognized [9, 10]. In major spinal surgeries, it has been proved to result in less blood loss and requirement of blood transfusion [11].

Controlled hypotension usually aimed at the reduction of the mean arterial pressure (MAP) by 30% during anesthesia and surgery, while still providing adequate oxygen delivery to vital organs [12]. This is usually achieved by an absolute systolic pressure drop to about 80–90 mmHg, which translates to a MAP of 50–65 mmHg [12]. Aging of the vascular system in elderly patients is often accompanied by increased vascular stiffness and decreased wall elasticity, which in turn leads to an increase in the proportion of simple systolic hypertension and microangiopathy, thereby affecting tissue perfusion [13]. Therefore, a low targeted MAP in controlled hypotension may be not suitable in these patients. An alternative is to combine controlled hypotension with higher targeted MAP with restrictive perioperative fluid supplement to ensure hemodynamic stability, improving perfusion and oxygenation of tissues and organs, as well as postoperative rehabilitation [14, 15]. It has been suggested that restrictive infusion in elderly patients reduced the incidence of postoperative anastomotic fistula and reduced the time of hospital stay [16].

However, restrictive transfusion infusion may lead to subclinical low circulatory blood volume, causing redistribution of systemic blood flow, leading to insufficient perfusion of the gastrointestinal tract, kidney, heart, and brain [17]. If controlled hypotension anesthesia is performed on the basis of restrictive transfusion, the additive effects on blood perfusion and systemic oxygen supply should be considered [8]. In addition, during controlled hypotension, renal blood flow decreases, and renal vasoconstriction, anti-diuretic hormone, and aldosterone secretion increase because of the drop in systemic perfusion pressure, which could lead to a decrease in glomerular filtration rate (GFR) [18].

Given these concerns, the effect of hypotension in combination with restrictive transfusion in elderly patients requires further investigation. Here, we explored the effects of controlled hypotension with two target MA*P* values (65 mmHg VS 75 mmHg) in combination with limited infusion, in elderly patients underwent lumbar fusion, in perspectives of intraoperative blood loss, systemic oxygen metabolism, and renal function.

## Methods

### Patients

In this exploratory interventional study, patients who were planned to undergo posterior lumbar fusion in Zhongda Hospital, Southeast University, during the period from March 2016 to January 2017, were screened. The inclusion criteria are (1) planned elective posterior multilevel (2–3) lumbar fusion under general anesthesia through tracheal intubation; (2) aged 60–75 years; (3) ASA grade II or III; and (4) estimated operative time > 120 min. The exclusion criteria were (1) preoperative blood pressure systolic ≥ 160 mmHg or diastolic ≥ 100 mmHg or pulse pressure ≥ 60 mmHg; (2) comorbidities of severe cardiovascular disease, including cardiac function grade III or IV, severe aortic stenosis, aortic valve insufficiency, severe coronary heart disease, bradycardia, and atrioventricular block above degree I; (3) history of cerebral infarction, Alzheimer’s disease, or cerebrovascular incident; (4) liver and/or kidney dysfunction; (5) severe anemia, shock, hypovolemia, or respiratory dysfunction; (6) previous history of phlebitis or thrombosis, closed-angle glaucoma; and (7) BMI > 25 or < 18. Patients with intraoperative blood loss > 1500 mL or operation time > 4 h would be further excluded from the analysis. This study was approved by the Ethics Committee of Zhongda Hospital, Southeast University (No. 2015ZDSYLL086.0). All patients gave written informed consent.

### Intervention

Patients were allocated into trial group (MAP 65 group) or control group (MAP 75 group) with simple randomization method using random number table.

All operations were performed under general anesthesia, induced by intravenous infusion of 0.05 mg/kg midazolam (Jiangsu Enhua Pharmaceutical Co., Ltd., China; 20151002), 0.2–0.3 μg/kg sufentanil (Yichang human welfare pharmaceutical Co., Ltd., China; 1150720), 1.5 mg/kg propofol (Beijing Fesenius Kabi Medical Co., Ltd.; 10IC8536), and 0.6 mg/kg rocuronium (Oganon Corp. China; 640822). After the mask was pressurized with oxygen for 2 min, endotracheal intubation was performed. Mechanical ventilation was set at tidal volume of 8 mL/kg and ventilation frequency of 10–12 breaths per min. The breathing ratio was set to 1.0:2.0, with pure oxygen flow rate of 2.0 L/min and maintained PETCO_2_ of 35~45 mmHg. After anesthesia induction, continuous infusion with propofol combined with sevoflurane (Marubeni pharmaceutical Co., Ltd.; 54171) and remifentanil was used to maintain analgesia, and intravenous injection of cis-atracurium (Jiangsu Hengrui Medical Co., Ltd., China; 15112317) was applied to maintain muscle relaxation.

### Controlled hypotension

After the circulation stabilized, blood pressure control was initiated in both groups at time of incision, with the MAP 65 group aiming at maintaining MAP of 65 mmHg and the MAP 75 group aiming at maintaining MAP of 75 mmHg. Nitroglycerin was intravenously administrated at initial rate of 0.5 g/kg/min using a programmed micropump. Blood pressure was continuously monitored; in every 3 min that the targeted MAP was not achieved, the infusion rate was increased by 0.5 μg/kg/min until it was reached. Nitroglycerin infusion would be terminated immediately in case that MAP drop below the targeted goal, and in case when the MAP was still lower than targeted 3 min after termination of nitroglycerin infusion, a low dose of noradrenaline (0.01~0.03 μg/kg/min) was applied to recover the target pressure. Blood pressure was controlled until the end of the decompression of the spinal canal.

During surgery, heart rate was controlled at 50–130 bpm, bispectral index (bis) was kept in anesthetic depth at 40–60, and body temperature was maintained at 36.3–37.2 °C. Importantly, if ischemic changes were observed on electrocardiogram, hypotension control was abandoned and this patient will be excluded from the study.

### Fluid management

Both groups were fasted before anesthesia. Lactated Ringer’s injection was administrated at 3 mL/kg/h. Intraoperative blood loss was recovered by autologous blood transfusion and supplement of hydroxyethyl starch 130/0.4 in 0.9% sodium chloride at 1:1 ratio.

### Outcomes and data collection

Parameters of perioperative bleeding, hemodynamic parameters, systemic oxygen metabolism, kidney functions, as well as procedure process and postoperative adverse reactions were recorded and compared between the groups.

Intraoperative dosage of crystal and colloid fluid, blood loss, volume, operation time of autologous blood transfusion, duration of hypotension, extubation time, exhaust time, and hospitalization time were recorded.

After patients entered the operation room, vital signs were monitored. Left radial artery catheters were inserted under local anesthesia. Flotrac sensors and Vigileo monitors were used to monitor MAP and cardiac index (CI). After endotracheal intubation, a catheter was inserted into the right internal jugular vein at a depth of 15 cm for both pump injection of nitroglycerin and monitoring of central venous pressure (CVP). Urinary catheterization was used to monitor urine volume.

The values of MAP, HR (heart rate), CI (cardiac index), and CVP (central venous pressure) before operation (T0), 1 h after hypotension (T1), 2 h after hypotension (T2), and in recovery stage (T3) were monitored and recorded.

Arterial and central venous blood samples were collected at the time points as mentioned above. Levels of hemoglobin (Hb), lactate (lac), arterial partial pressure of oxygen (PaO_2_), central venous oxygen partial pressure (PcVO_2_), arterial oxygen saturation (SaO_2_), central venous oxygen saturation (ScVO_2_), oxygen supply index (DO_2_I), oxygen consumption index (VO_2_I), and oxygen uptake rate (ERO_2_) were detected using blood gas analyzer (NOVA Ultra; Nova Inc., USA).

Postoperative adverse reactions monitored included pulmonary complications (cough, sputum, shortness of breath, or dyspnea; pulmonary infection signs in chest X-ray imaging; or atelectasis), gastrointestinal complications (nausea, vomiting, abdominal distension, or bowel sound disappearance), and wound infection (aggravated pain at wound position, local redness and tenderness, blood oozing and exudate on dressing, and unsatisfied wound healing).

Basic clinical data were collected from the patients’ electronic medical records.

### Statistical analysis

Quantitative data were expressed as means ± SD and categorical data were expressed as frequency (percentage. The Shapiro–Smirnov test (*d* test) was used to test the normality of data distribution. The independent two samples Student *t* test was used for the comparison between normally distributed quantitative data between the groups, and skewed distributed data were compared using two samples Mann–Whitney *U* test. The *χ*^2^ test was used to compare categorical data. A *p* value of < 0.05 was considered statistically significant. All statistical analyses were performed using SPSS version 22.0 (IBM Corp., Armonk, NY, USA).

## Results

### Patients

**Fig. 1:**
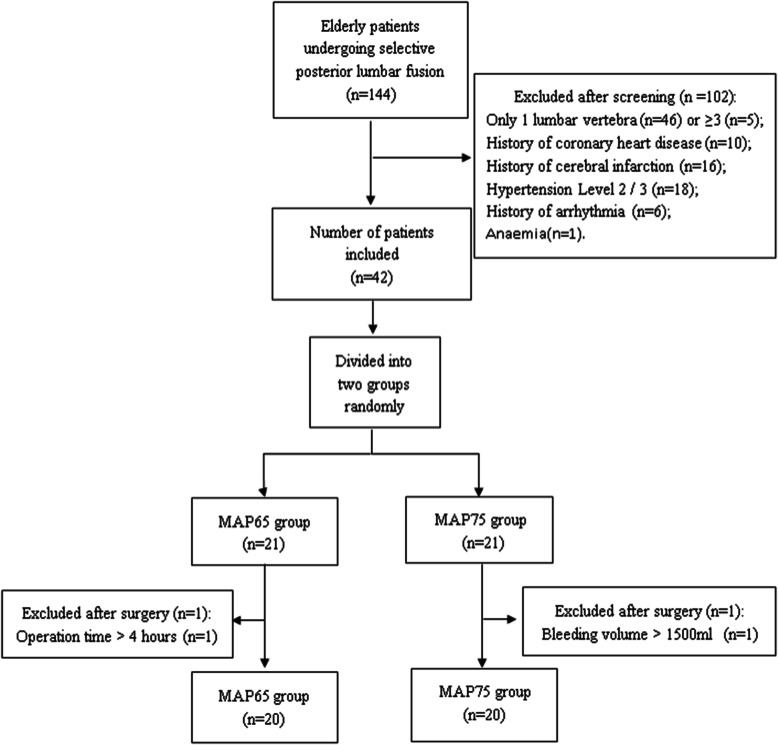
Flowchart of inclusion and exclusion

A total of 144 elderly patients were scheduled to undergo selective posterior lumbar fusion during the study period, with 102 cases were excluded for different reasons. In addition, one patient in the MAP 65 group for whom the operation time was more than 4 h and one case in the MAP 75 group with intraoperative blood loss larger than 1500 ml were excluded, resulting in 40 patients, 20 in each group, in the final analysis (Fig. 1). Patients in the two groups showed no significant difference in age, sex, lumbar space, operation time, anesthetic time, or duration of hypotension (Table 1).

**Table 1:** Comparison of general data and surgical-related indexes between the two groups

	MAP 65 group *n* = 20	MAP 75 group *n* = 20	*P* value
Age (years)	67 ± 5	68 ± 6	0.9537
Gender (M/F)	7/13	11/9	0.3406
BMI (kg/m2)	21.9 ± 1.9	22.5 ± 1.5	0.2643
ASA Grading composition (II/III)	14/6	15/5	> 0.9999
Lumbar vertebrae (2/3)	14/6	13/7	> 0.9999
operation time (min)	196 ± 32	195 ± 34	0.9393
Anesthesia duration (min)	231 ± 33	226 ± 34	0.6397
BP control time (min)	129 ± 11	130 ± 12	0.7850

### Comparison of blood loss between the two groups

As shown in Table 2, the amount of bleeding was not different between the two groups of patients (*p* > 0.05), and all patients did not receive allogeneic blood transfusion during surgery. There was neither significant difference in urine volume, colloidal fluid dosage, crystalloid dosage, total fluid volume, and auto-blood reclaiming between the two groups (Table 2).

**Table 2:** Comparison of intake and output between the two groups

	MAP 65 group *n* = 20	MAP 75 group *n* = 20	*P* value
Amount of bleeding (ml)	493 ± 294	564 ± 311	0.4627
Urine volume (ml)	259 ± 123	210 ± 98	0.1716
Colloidal fluid usage (ml)	215 ± 121	210 ± 140	0.9045
Crystal liquid usage (ml)	1160 ± 238	1135 ± 181	0.7105
Total amount of liquid (ml)	1392 ± 263	1350 ± 221	0.5877
Auto-blood reclaiming (ml)	313 ± 255	388 ± 56	0.2067

### Comparison of hemodynamic indicators between the two groups

**Fig. 2:**
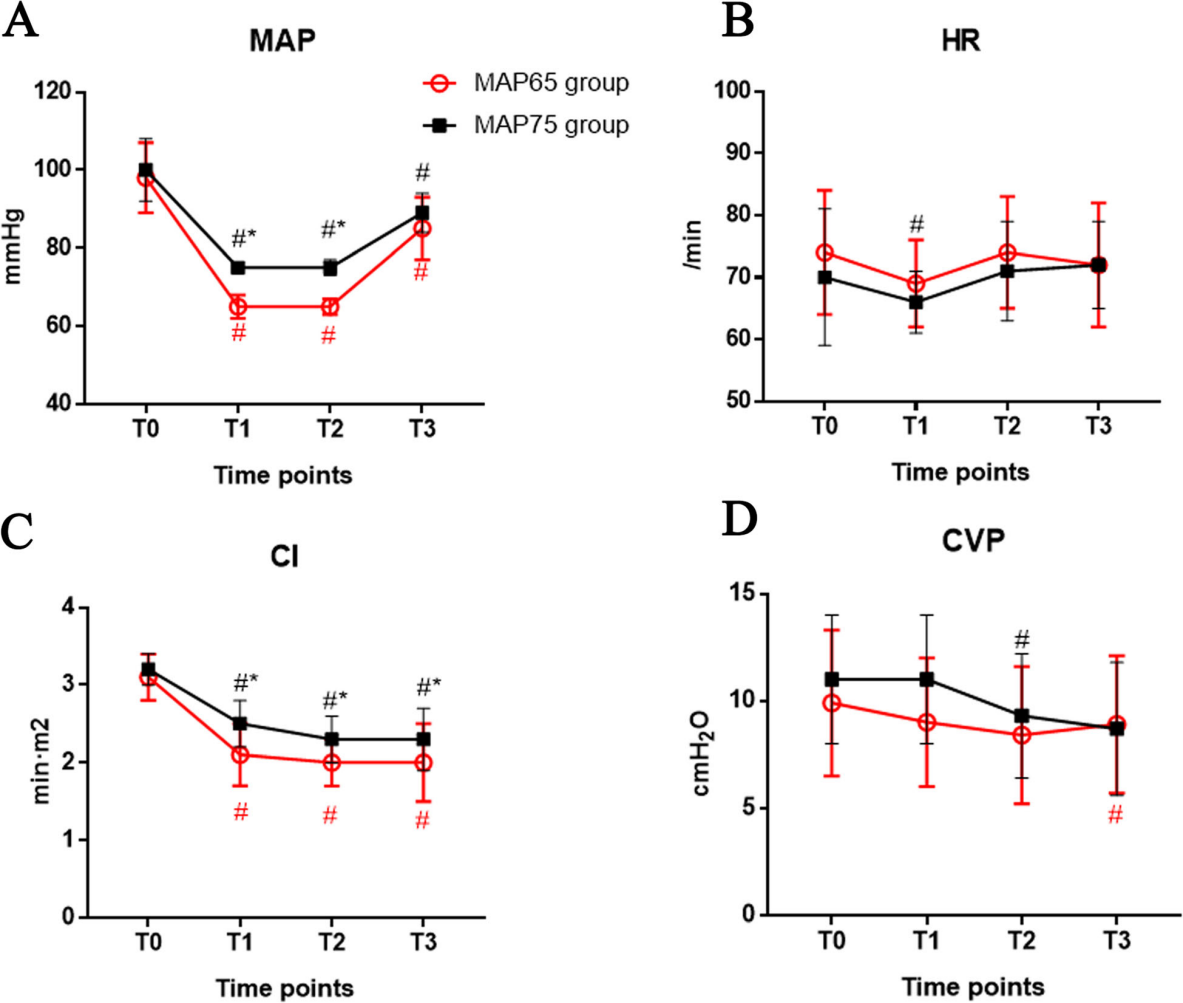
Comparison of hemodynamic change between the two groups

As shown in Table 3 and Fig. 2, there was no significant difference in MAP between the two groups at T0 (*P* > 0.05). Both groups were able to maintain targeted MAP at T1 and T2. In addition, MAP during recovery period (T3) was significantly decreased compared to T0 (*P* < 0.05) (Fig. 2a).

**Table 3:** Comparison of hemodynamic change between the two groups

	Group	T0	T1	T2	T3
MAP (mmHg)	MAP 65 group	98 ± 9	65 ± 3^#*^	65 ± 2^#*^	85 ± 8^#^
	MAP 75 group	100 ± 8	75 ± 1^#^	75 ± 2^#^	89 ± 5^#^
HR (/min)	MAP 65 group	74 ± 10	69 ± 7^#^	74 ± 9	72 ± 10
	MAP 75 group	70 ± 11	66 ± 5	71 ± 8	72 ± 7
CI (L/(min m^2^))	MAP 65 group	3.1 ± 0.3	2.1 ± 0.4^#*^	2.0 ± 0.3^#*^	2.0 ± 0.5^#*^
	MAP 75 group	3.2 ± 0.2	2.5 ± 0.3^#^	2.3 ± 0.3^#^	2.3 ± 0.4^#^
CVP (cmH_2_O)	MAP 65 group	9.9 ± 3.4	9.0 ± 3.0	8.4 ± 3.2^#^	8.9 ± 3.2
	MAP 75 group	11.0 ± 3.0	11.0 ± 3.0	9.3 ± 2.9	8.7 ± 3.1^#^

Compared with T0, ^#^*P* < 0.05; compared with MAP 75 group, *P < 0.05

Before surgery (T0), there was no difference in HR between the two groups. In the MAP 65 group, significant decrease of HR compared to T0 was observed at T1 (*P* < 0.05), but not at T2 or T3 (*P* > 0.05). In the MAP 75 group, no significant changes in HR compared to that at T0 for all observed time points (*P* > 0.05). In addition, at all time points examined, HRs were not different between the two groups (*P* > 0.05) (Fig. 2b).

There was no significant difference in CIs between the two groups at T0 (*P* > 0.05). Compared to T0, CIs in both groups decreased significantly at T1–T3 (*P* < 0.05). Importantly, the CIs of the MAP 65 group were significantly lower than those of the MAP 75 group at T1–T3 (*P* < 0.05) (Fig. 2c).

Similarly, there was no significant difference in CVPs between the two groups at T0 (*P* > 0.05). In the MAP 65 group, significant decrease of CVP compared to T0 was observed at T2 (*P* < 0.05), but not at T1 and T3 (*P* > 0.05). In the MAP 75 group, CVPs at T1 and T2 were not significantly different from that of T0 (*P* > 0.05), but significantly lower CVP at T3 compared to T0 was observed (P < 0.05) (Fig. 2d).

### Comparison of systemic oxygen metabolism between the two groups

There were no significant differences in PaO_2_, SaO_2_, PcvO_2_, and ScvO_2_ between the two groups at any time point (*P* > 0.05). In addition, no significant changes during and after procedure were observed in these parameters compared to T0 in both the two groups (*P* > 0.05) (Table 4).

**Table 4:** Comparison of changes in systemic oxygen metabolism between the two groups ($\overline{\mathrm{x}}$ ± *s*, *n* = 20)

	Group	T0	T1	T2	T3
PaO_2_ (mmHg)	MAP 65 group	393 ± 118	391 ± 118	426 ± 84	430 ± 105
	MAP 75 group	419 ± 86	431 ± 87	436 ± 94	418 ± 98
SaO_2_ (%)	MAP 65 group	99.6 ± 0.5	99.6 ± 0.4	99.6 ± 0.3	99.6 ± 0.3
	MAP 75 group	99.4 ± 1.2	99.8 ± 0.1	99.8 ± 0.1	99.7 ± 0.1
PcvO_2_ (mmHg)	MAP 65 group	55 ± 9	60 ± 9	59 ± 10	59 ± 8
	MAP 75 group	55 ± 5	56 ± 6	57 ± 8	59 ± 5
ScvO_2_ (%)	MAP 65 group	85.3 ± 5.1	85.7 ± 5.0	84.7 ± 5.2	85.2 ± 4.1
	MAP 75 group	85.7 ± 3.3	84.9 ± 3.5	84.8 ± 4.9	86.3 ± 4.9
CaO_2_ (ml/L)	MAP 65 group	163 ± 8	159 ± 10	152 ± 14^#^	154 ± 14^#^
	MAP 75 group	166 ± 10	161 ± 11^#^	156 ± 12^#^	159 ± 10^#^
CcvO_2_ (ml/L)	MAP 65 group	137 ± 10	136 ± 13	129 ± 18^#^	131 ± 14
	MAP 75 group	142 ± 10	136 ± 12^#^	131 ± 13^#^	136 ± 10
Hb (g/L)	MAP 65 group	121 ± 6	119 ± 7	113 ± 10^#^	115 ± 10^#^
	MAP 75 group	123 ± 8	120 ± 8^#^	115 ± 9^#^	118 ± 7^#^
Lactic acid (mmol/L)	MAP 65 group	1.2 ± 0.5	1.0 ± 0.3	1.0 ± 0.4	1.0 ± 0.5
	MAP 75 group	1.0 ± 0.5	0.9 ± 0.4	0.9 ± 0.4	0.9 ± 0.4
DO_2_I (ml/min m^2^)	MAP 65 group	509 ± 57	331 ± 62^#*^	310 ± 61^#*^	311 ± 82^#*^
	MAP 75 group	524 ± 45	405 ± 47^#^	349 ± 56^#^	366 ± 61^#^
VO_2_I (ml/min m^2^)	MAP 65 group	78 ± 18	48 ± 19^#*^	47 ± 14^#^	47 ± 18^#^
	MAP 75 group	76 ± 21	63 ± 15^#^	55 ± 19^#^	52 ± 21^#^
ERO_2_	MAP 65 group	0.15 ± 0.04	0.15 ± 0.05	0.16 ± 0.05	0.15 ± 0.04
	MAP 75 group	0.14 ± 0.03	0.16 ± 0.03	0.16 ± 0.05	0.14 ± 0.05

Compared with T0, ^#^*P* < 0.05; compared with MAP 75 group, *P < 0.05

The levels of CaO_2_ and CcVO_2_ were not different between the two groups at each time point (*P* > 0.05). In the MAP 65 group, compared with T0, the decrease in CaO_2_ at T2 and T3 was statistically significant (*P* < 0.05), and CcVO_2_ decreased significantly at time T2 (*P* < 0.05). In the MAP 75 group, the decrease in CaO_2_ at T1–T3 time was statistically significant (*P* < 0.05), and CcVO_2_ decreased significantly at the T1 and T2 (*P* < 0.05) compared to T0.

There was no significant difference in [Hb] s between the two groups at all time points (*p* > 0.05). There was no significant difference in [Hb] between T2 and T3 and between T1 and T0 (*p* < 0.05). At T1–T3 time points, Hb concentration decreased in the MAP 75 group (*p* < 0.05). Interestingly, lactic acid concentration did not change over time in either the MAP 65 group or MAP 75 group.

At T0, the oxygen delivery index (DO_2_I) and oxygen consumption index (VO_2_I) was not different between the two groups. In both groups, DO_2_I and VO_2_I decreased in T1–T3 (*p* < 0.05) compared with T0. Meanwhile, the DO_2_I in the MAP 65 group at T1–T3 were lower than that in the MAP 75 group. Oxygen extraction rate (ERO_2_) did not change in the process.

### Comparison of renal function parameters between the two groups

There were no differences between the two groups in serum cystatin C (SCysC), creatinine (Cr), and BUN at different time points. In the MAP 65 group, compared with T0, the level of SCysC decreased in T1–T3, Cr decreased in T2, but BUN had no changes. In the MAP 75 group, compared with T0, the level of SCysC increased at T2 and decreased at T4, the level of Cr increased at T1 and T3, and BUN increased at T1 and T4 (Table 5).

**Table 5:** Comparison of renal function indexes between two groups at different time points ($\overline{\mathrm{x}}$ ± *s*, *n* = 20)

	Group	T0	T1	T2	T3	T4
SCysC (mg/L)	MAP 65 group	0.74 ± 0.13	0.81 ± 0.13^#^	0.87 ± 0.14^#^	0.79 ± 0.14^#^	0.71 ± 0.13
	MAP 75 group	0.76 ± 0.16	0.80 ± 0.17	0.84 ± 0.18^#^	0.84 ± 0.20	0.68 ± 0.13^#^
Cr (μmol/L)	MAP 65 group	76 ± 15	78 ± 14	80 ± 13^#^	74 ± 14	77 ± 14
	MAP 75 group	72 ± 13	76 ± 16^#^	74 ± 21	79 ± 15^#^	72 ± 14
BUN (mmol/L)	MAP 65 group	4.8 ± 0.9	4.9 ± 1.0	5.2 ± 1.5	5.2 ± 1.3	4.5 ± 0.8
	MAP 75 group	4.6 ± 1.0	4.7 ± 1.0^#^	4.8 ± 1.3	4.8 ± 1.0	4.1 ± 1.3^#^

Compared with T0, ^#^*P* < 0.05; compared with MAP 75 group, *P < 0.05

### Comparison of postoperative adverse reactions between the two groups

There was no difference in extubation time or hospitalization time between the two groups. Importantly, postoperative fever, wound infection, pulmonary complications, and gastrointestinal complications were not significantly different between the MAP 65 group and MAP 75 group (Table 6).

**Table 6:** Comparison of renal function indexes between two groups at different time points ($\overline{\mathrm{x}}$ ± *s*, *n* = 20)

	MAP 65 group (*n* = 20)	MAP 75 group (*n* = 20)	*P* value
Extubation time (min)	19 ± 9	23 ± 13	0.2650
Hospitalization time (days)	13 ± 3	14 ± 3	0.2985
Postoperative fever (%)	20	30	0.7164
Wound infection (%)	0	0	> 0.9999
Pulmonary complications (%)	0	0	> 0.9999
Gastrointestinal complications (%)	5	10	> 0.9999

## Discussion

In this study, a pilot interventional study was performed to evaluate whether controlled hypotension in combination with restrictive transfusion can improve systemic oxygen metabolism, reduce intraoperative bleeding, and improve systemic hemodynamics. The results showed that controlled hypotension in combination with restrictive transfusion may improve renal function, reduce the risk of insufficient oxygen supply, and does not result in increased intraoperative blood loss or increased postoperative complications.

Previous study has shown that controlled hypotension reduces peripheral vascular resistance, resulting in reduced blood leakage from subcutaneous tissue, paravertebral muscles, and epidural veins [19]. Wonjung et al. found that the blood loss of the elderly (aged 60–70) patients who underwent spinal surgery without controlled hypotension was 765 ± 339 mL, while turned to 445.0 ± 226.5 mL with controlled hypotension [6]. In this study, the amount of intraoperative blood loss was 493 ± 294 mL and 564 ± 311 mL in the two groups. Although blood loss volumes in different studies may not be comparable, the results still may hint that induced hypotension in this study could have resulted in less blood loss. There was little need for transfusion of allogeneic blood. Although no statistical significant difference was detected between the two groups, which may be due to the limited sample size, the results did indicate an even lower amount of blood loss when MAP was controlled at 65 mmHg with restrictive infusion, compared to targeted MAP of 75 mmHg.

Controlled hypotension, as well as surgical posture itself, may have a significant effect on CI. It has been suggested that the protruding position pad caused a significant decrease in CI, but no significant change in other hemodynamic indexes [20]. In this study, CI in both groups decreased significantly compared with t0 at T1–T3 time point, which indicates that the decrease in blood pressure will further reduce CI, in addition to the postural change. The results of CVP changes were not expected, especially when MAP was controlled at 75 mmHg. We suspect that the prone position affected the vena cava reflux, thereby increasing CVP. Furthermore, even under the condition of controlled hypotension and restrictive infusion, the change in CVP was not significant and whether it is a repeatable phenomenon requires further study.

The purpose of monitoring hemodynamic indexes during operation is to maintain good perfusion of tissues and organs to maintain normal metabolism and prevent hypoxia and apoptosis of tissue cells from causing dysfunction of the organism. The most basic clinical monitoring indexes such as SpO_2_ and PetCO_2_ cannot fully describe the state of oxygen metabolism. At present, the main indexes of oxygen metabolism are oxygen supply index (DO_2_I), oxygen consumption index (VO_2_I), oxygen uptake rate (ERO_2_), and lactate. In this experiment, the two groups were both on pure oxygen positive pressure ventilation, and there was no significant difference in Hb, PaO_2_, SaO_2_, and CaO_2_; therefore, the decrease in DO_2_I might be mainly due to the decrease in CI. In addition, the decreasing trends of CaO_2_ and [Hb] were consistent, so the changes of DO_2_I were mainly affected by [Hb] and CI over time. All these results suggested that the combination of controlled hypotension and restrictive transfusion could significantly improve oxygen supply.

In this study, renal function indicators including urea nitrogen (BUN), creatinine (Cr), and serum cystatin (SCysC) were dynamically monitored. Compared with T0, SCysC in the MAP 65 group increased significantly after hypotension and recovered to the initial level 1 day after operation, whereas Cr only increased significantly at T2. SCysC in the MAP 75 group only increased significantly at T2, Cr rises at T1 and T3, and BUN increases at T1 and decreases at T4. These results indicated that in elderly patients, reducing MAP to 65 mmHg while limiting fluid intake may result in a slight improvement of renal function. Whether GFR can be reduced effectively or not, as well as the obvious improvement of renal function, remains to be further studied.

Our study has potential limitations. First, in order to strictly adhere to inclusion and exclusion criteria, a limited number of cases were selected, resulting in a smaller sample size. Second, chronic diseases which are common in elderly may affect the changes in the monitoring parameters while due to limited sample size and data, the influences were not explored. Further research needs to be explored in this regard.

## Conclusions

In elderly patients subjected to posterior lumbar fusion, controlled hypotension targeting a MAP of 75 mmHg in combination with restrictive transfusion, compared with controlled hypotension with 65 mmHg, met the requirements for reducing intraoperative blood loss and allogeneic blood transfusion, with little effects on renal function and oxygen supply, whereas control hypotension with targeted MAP of 65 mmHg may significantly reduce the risk of CI and oxygen supply.

## Data Availability

All data generated or analyzed during this study are included in this published article.

## Abbreviations

DDD: Degenerative disk diseases
MAP: Mean arterial pressure
bis: Bispectral index
CI: Cardiac index
CVP: Central venous pressure
Hb: Hemoglobin
lac: Lactate
PaO_2_: Arterial partial pressure of oxygen
PcVO_2_: Central venous oxygen partial pressure
SaO_2_: Arterial oxygen saturation
ScVO_2_: Central venous oxygen saturation
DO_2_I: Oxygen supply index
VO_2_I: Oxygen consumption index
ERO_2_: Oxygen uptake rate
DO_2_I: Oxygen delivery index
ERO_2_: Oxygen extraction rate
SCysC: Serum cystatin C
Cr: Creatinine
BUN: Urea nitrogen
GFR: Glomerular filtration rate
